# Lactoferrin in Pediatric Chronic Kidney Disease and Its Relationship with Cardiovascular Risk

**DOI:** 10.3390/children11091124

**Published:** 2024-09-13

**Authors:** Chun-Yi Ho, Pei-Chen Lu, Wei-Ling Chen, Wei-Ting Liao, Chien-Ning Hsu, You-Lin Tain

**Affiliations:** 1 Department of Pediatrics, Kaohsiung Chang Gung Memorial Hospital, Kaohsiung 833, Taiwan; insomniac@cgmh.org.tw (C.-Y.H.); latina@cgmh.org.tw (P.-C.L.); weilingchen@cgmh.org.tw (W.-L.C.); winona0409@cgmh.org.tw (W.-T.L.); 2 Department of Pediatrics, Kaohsiung Municipal Feng Shan Hospital—Under the Management of Chang Gung Medical Foundation, Kaohsiung 830, Taiwan; 3 School of Pharmacy, Kaohsiung Medical University, Kaohsiung 807, Taiwan; cnhsu@cgmh.org.tw; 4 Department of Pharmacy, Kaohsiung Chang Gung Memorial Hospital, Kaohsiung 833, Taiwan; 5 College of Medicine, Chang Gung University, Taoyuan 330, Taiwan; 6 Institute for Translational Research in Biomedicine, Kaohsiung Chang Gung Memorial Hospital, Kaohsiung 833, Taiwan

**Keywords:** chronic kidney disease, biomarker, hypertension, cardiovascular disease, lactoferrin, ambulatory blood pressure monitoring, children

## Abstract

Background: Pediatric CKD is associated with a high risk of cardiovascular disease (CVD). Early detection of subclinical CVD in childhood CKD can be achieved through various cardiovascular (CV) assessments, including carotid intima–media thickness (cIMT), ambulatory blood pressure monitoring (ABPM), and arterial stiffness indices. Lactoferrin (LF), a key functional glycoprotein found in breast milk, has been linked to several diseases and has potential as a biomarker. Methods: In our study of 102 children with CKD stages G1–G4, we explored the relationship between LF and CV risk markers. Results: We found that LF concentration was not related to the severity or underlying causes of childhood CKD, but was positively correlated with overweight/obesity. Lower LF levels were correlated with increased cIMT and elevated arterial stiffness indices. Notably, abnormalities in ABPM profiles were observed in up to 60% of the children with CKD, with low LF levels linked to nighttime hypertension, nocturnal non-dipping, and ABPM abnormalities. Conclusions: In conclusion, LF shows promise as a biomarker for detecting subclinical CVD in children with CKD. Its potential utility in early detection could be instrumental in guiding timely interventions and improving long-term CV outcomes, although further research is needed to clarify the underlying mechanisms.

## 1. Introduction

Chronic kidney disease (CKD) is an escalating global public health challenge affecting both adults and children [1,2]. Since adult CKD may originate in early life [3], it is crucial to concentrate on pediatric CKD, with a particular emphasis on accurate prediction and optimal care for high-risk children [4]. CKD is accompanied by a high risk of cardiovascular disease (CVD) [5]. Although overt CVD is uncommon in children, the underlying process of atherosclerosis can start at a young age. Currently, numerous biomarkers have been identified in CKD, reflecting either the pathogenesis of CKD progression or cardiovascular risk [6,7]. However, their validation in pediatric CKD remains very limited [8]. 

Proteomic mass spectrometry has significantly advanced the identification of protein biomarkers across various etiologies of CKD [9]. Our research team previously proposed investigating whether proteomic biomarkers of cardiovascular risk in childhood CKD could be derived [9]. We identified 20 proteins with differential expression linked to hypertension in children with CKD, some of which were validated in subsequent experiments [10,11]. Building upon this previous research [10], we evaluated lactoferrin in a pediatric CKD cohort to explore its association with CKD-related complications.

Lactoferrin (LF), a multifunctional glycoprotein found in the breast milk of most mammals, possesses a variety of biological activities, such as antioxidant, antiviral, anti-inflammatory, and prebiotic effects [12]. Increasing evidence supports the positive health impact of LF on infants and children [13]. Conversely, abnormal levels of LF are associated with various diseases, making LF a potential biomarker for conditions such as inflammatory bowel disease, dry eye disease, and Alzheimer’s disease [14]. In the kidneys, LF has been reported to reduce apoptosis, inflammation, oxidative stress, and kidney fibrosis. However, research on LF as a biomarker in kidney disease is currently lacking [15,16].

Several recently reported noninvasive cardiovascular assessments, such as carotid intima–media thickness (cIMT) [17], 24 h ambulatory blood pressure monitoring (ABPM) [17], pulse wave velocity (PWV) [18], and left ventricular mass index (LVMI) [19], are accessible for estimating the risk for CVD in childhood CKD. Therefore, our study aimed to examine the associations between plasma LF concentration and ABPM and other CVD surrogate markers (i.e., cIMT, PWV, and LVMI) in children with CKD. 

## 2. Materials and Methods

### 2.1. Study Population and Setting

This cross-sectional study was performed at Kaohsiung Chang Gung Memorial Hospital, Taiwan, and was approved by the ethics committee (201701735A3C501 and 202001973A3C601). A total of 102 children and adolescents with CKD stages 1–4 who received the measurement of LF were recruited from a prospective cohort study. The inclusion criteria encompassed youths aged 3–18 years with CKD stages G1–G4, as defined by KDIGO [20]. For adolescents over 16 years of age, estimated glomerular filtration rates (eGFRs) were calculated using the EPI-CKD equation. For children aged 2–16 years, the modified Schwartz pediatric bedside formula was used [21]. Children with CKD and an eGFR ≥ 90 mL/min/1.73 m^2^ were classified as stage G1. Stage G2 corresponds to an eGFR of 60–89 mL/min/1.73 m^2^, stage G3 to 30–59 mL/min/1.73 m^2^, and stage G4 to 15–29 mL/min/1.73 m^2^. Exclusion criteria included pregnancy or lactation, CKD stage G5, kidney transplant, current dialysis, congenital heart disease, and lack of patient cooperation.

The causes of CKD were categorized based on whether or not congenital anomalies of the kidney and urinary tract (CAKUT) were present. CAKUT includes conditions such as kidney hypoplasia/dysplasia, kidney agenesis, horseshoe kidney, duplex collecting system, multicystic dysplastic kidney, posterior urethral valves, and ureteral abnormalities [22]. Non-CAKUT etiologies of CKD include glomerular diseases, nephrotic syndrome, tubulointerstitial diseases, renal artery stenosis, lupus nephritis, and inherited metabolic disorders, such as Fanconi syndrome.

After an overnight fast, peripheral venous blood was collected, promptly refrigerated, and stored at −80 °C. Glucose, blood urea nitrogen, creatinine, urine total protein-to-creatinine ratio, total cholesterol, triglyceride, low-density lipoprotein (LDL), sodium, potassium, uric acid, calcium, phosphate, hemoglobin, and hematocrit levels were investigated by the central laboratory at our hospital, as stated previously [10,11]. Hyperuricemia was diagnosed based on serum uric acid levels exceeding 5.9 mg/dL in 3- to 8-year-olds, 6.1 mg/dL in 9- to 11-year-olds, 7.0 mg/dL in males aged 12 years and older, or 6.2 mg/dL in females aged 12 years and older [23]. Hyperlipidemia was defined based on serum lipid levels: total cholesterol ≥ 200 mg/dL, LDL cholesterol ≥ 130 mg/dL, or triglycerides ≥ 100 mg/dL in children aged 0 to 9 years. For adolescents aged 10 to 19 years, the threshold for hyperlipidemia was set at triglycerides ≥ 130 mg/dL [24]. Obesity and overweight were defined as a body mass index (BMI) percentile ≥ 95th and ≥85th percentile, respectively. BMI was determined by dividing body weight in kilograms by the square of height in meters. BMI percentiles for age and sex were determined using guidelines from the Health Promotion Administration at the Ministry of Health and Welfare in Taiwan [25].

### 2.2. Cardiovascular Assessment

Office BP was measured using an oscillometric device with a correctly sized cuff while the child was seated calmly with their arm supported at heart level. Hypertension was defined based on the 2017 AAP guidelines [26]. Out of 102 participants, 62 children aged over 6 years underwent cardiovascular assessment and ABPM analysis.

ABPM was conducted using the Oscar II monitoring device (SunTech Medical, Morrisville, NC, USA) with appropriately sized cuffs, as previously described [10,11]. ABPM abnormalities included: (1) systolic, diastolic, awake, or asleep BP loads ≥ 95th percentile for height and gender; (2) systolic, diastolic, awake, or asleep BP load ≥ 25%; and (3) non-dipping patterns in systolic or diastolic BP, characterized by a dipping percentage of less than 10% [27]. 

The ambulatory arterial stiffness index (AASI) is an indirect arterial stiffness index that has been shown to be linked with adverse cardiovascular events [28]. The AASI was computed from 24-h ABPM by calculating the regression slope of diastolic BP on systolic BP [29].

Using a Philips IE33 cardiac ultrasound (Bothell, WA, USA), the left ventricular mass (LVM) was measured, and the LVM index (LVMI) was calculated by normalizing LV mass to height^2.7^ [30]. Carotid ultrasound was conducted with an ALOKA ProSound α7 (Aloka Co., Tokyo, Japan) to assess carotid intima–media thickness (cIMT). Augmentation index and PWV were investigated using the e-TRACKING system.

### 2.3. Determination of Lactoferrin Concentration

The LF concentrations in the plasma were analyzed in duplicate using an ELISA assay (ab200015, Abcam, Cambridge, UK). Optical density was assessed at 450 nm, following the manufacturer’s instructions. The coefficient of variation was <15%.

### 2.4. Statistical Analysis

Descriptive statistics are described as medians and interquartile ranges (or ranges) for continuous variables, while categorical variables are expressed as numbers and percentages. Between-group differences were assessed for significance using the unpaired Mann–Whitney U test, Fisher’s exact test, and the chi-square test, as appropriate. Spearman rank correlation was utilized to test for associations. Multiple regression analysis revealed independent risk factors. A *p*-value < 0.05 was considered statistically significant. Statistical elaboration was performed using SPSS software 22.0 (Chicago, IL, USA).

## 3. Results

### 3.1. Cohort Characteristics

Table 1 provides an overview of the study participants’ characteristics. The cohort comprised 102 youths with CKD stages G1–G4. The median age of the participants was 9.97 years, with 54.9% being male and 65.7% having CAKUT. Most participants (71.6%, n = 73) were in CKD stage G1, followed by stage G2 (19.6%, n = 20), stage G3 (6.9%, n = 7), and stage G4 (2%, n = 2). This suggests that most of our study population was in the early stages of CKD.

Hypertension was diagnosed in 38.2% (n = 39) of the participants based on their office BP readings. Additionally, the prevalence of comorbidities among the participants included overweight/obesity in 34.3% (n = 35), hyperlipidemia in 33.3% (n = 34), hyperuricemia in 19.6% (n = 20), and proteinuria in 24.5% (n = 25).

Comparative analysis revealed that children in stages G2–G4 exhibited a male predominance, elevated systolic BP, and increased plasma concentrations of uric acid compared to those in stage G1.

### 3.2. Plasma Lactoferrin Concentration

**Figure 1:**
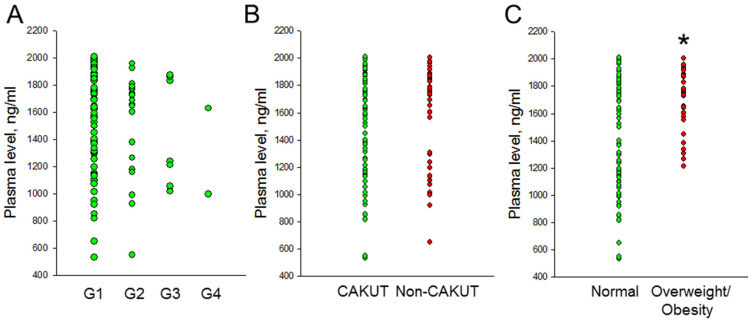
Comparison of plasma lactoferrin concentrations between (**A**) the CKD stages G1–G4 group, between (**B**) the CAKUT and non-CAKUT group, and between (**C**) the normal and overweight/obesity group. * *p* < 0.05 by the Mann–Whitney U-test.

We analyzed the plasma concentrations of LF in children and adolescents with CKD. Figure 1A illustrates that LF concentrations were comparable across different stages of CKD. No significant difference was observed in plasma LF concentrations between the CAKUT and non-CAKUT groups (*p* = 0.47). However, significant differences in LF concentrations were noted according to the presence or absence of overweight/obesity (Figure 1C, 1727 ± 221 vs. 1453 ± 402 ng/mL, *p* < 0.001). There was no significant association between LF and plasma Cr levels (*p* = 0.6) or eGFR (*p* = 0.302). Additionally, LF did not show a significant association with systolic BP (*p* = 0.104) or diastolic BP (*p* = 0.988), but it did show a positive correlation with BMI (r = 0.314, *p* = 0.001). 

### 3.3. Cardiovascular Assessment

In a subset of 62 children with CKD, a thorough cardiovascular evaluation was conducted concurrently (Table 2). We found that the LV mass, LVMI, and PWV were significantly higher in individuals with overweight/obesity compared to those without. However, no significant differences were found between the two groups regarding cIMT, augmentation index, and AASI.

Spearman’s rank correlation analysis identified associations between plasma LF concentrations and CV markers, as detailed in Table 3. LF level was negatively correlated with cIMT (*r =* −0.262, *p* = 0.04), augmentation index (*r =* −0.257, *p* = 0.043), and AASI (*r =* −0.357, *p* = 0.004). In multiple regression analysis for the AASI (F value = 3.334, *p* = 0.01), LF was identified as an independent risk factor (β = −0.364, *p* = 0.006), after adjusting for age, sex, overweight/obesity, and eGFR.

### 3.4. Plasma LF Concentration vs. ABPM Profile

Table 4 shows that 62.9% of the children (39/62) had at least one abnormality on ABPM. Specifically, 8 cases (12.9%) had 24-h BP ≥95th percentile, 9 cases (14.5%) had awake BP ≥ 95th percentile, 22 cases (35.5%) had asleep BP ≥95th percentile, 26 cases (41.9%) had BP load ≥ 25th percentile, and 33 cases (53.2%) had a nocturnal BP load decrease of less than 10%. Importantly, more than half of the children with CKD stage 1 (25 out of 44, or 56.8%) had at least one abnormality in their ABPM analysis. Additionally, plasma LF concentration was significantly lower in CKD children accompanied by nighttime hypertension (*p* = 0.015), nocturnal non-dipping (*p* = 0.004), and an ABPM profile that exceeded the target values (*p* = 0.006). Moreover, in the adjusted model controlling for age, sex, overweight/obesity, and eGFR, LF was associated with daytime hypertension (β = −0.003, *p* = 0.027), nighttime hypertension (β = −0.002, *p* = 0.034), nocturnal non-dipping (β = −0.002, *p* = 0.023), and an abnormal ABPM profile (β = −0.002, *p* = 0.024).

## 4. Discussion

To the best of our knowledge, this study represents the first investigation into LF as a potential protein biomarker for CVD in pediatric CKD. Our key findings are as follows: (1) Plasma LF concentration was not associated with the severity or underlying causes of childhood CKD but was positively correlated with BMI. (2) LF levels showed a negative correlation with cIMT, augmentation index, and AASI in children with CKD. (3) Abnormalities in the ABPM profile were present in up to 60% of children with CKD. (4) Low LF concentrations were observed in CKD children with nighttime hypertension, nocturnal non-dipping, and abnormal ABPM profiles. 

Consistent with previous research revealing that CVD risk markers can emerge early in pediatric CKD [5,6,7,8], including abnormalities detected through ABPM [31], our study found that 62.9% of children with CKD had ambulatory hypertension diagnosed through ABPM. In contrast, when hypertension was assessed using office BP measurements, only 38.2% of these children were found to have hypertension. This discrepancy highlights the significant prevalence of abnormalities detected by ABPM and supports the notion that ABPM may provide a more accurate and comprehensive assessment of hypertension and cardiovascular risk in children with CKD compared to traditional office BP measurements. This suggests that ABPM could be a crucial tool for the early detection and management of hypertension in this vulnerable population, potentially leading to better risk prediction and more effective intervention strategies [31]. 

Animal studies suggest that LF may offer protective effects against CKD [32], but its role as a biomarker in CKD remains unclear. LF concentrations at various stages of human CKD have not yet been reported. Our investigation revealed that LF concentration was not associated with the severity or underlying causes of childhood CKD, but was positively correlated with BMI. While some studies have reported an inverse correlation between plasma LF levels and overweight/obesity [33,34], the relationship between LF and BMI remains inconclusive [34,35]. The precise mechanisms underlying changes in plasma LF levels in the context of childhood obesity are not yet fully understood [36]. Given LF’s role in weight loss and glycolipid metabolism regulation, the elevated plasma LF levels observed in overweight/obesity may represent a compensatory response aimed at mitigating weight gain during the early stages of CKD.

Our results revealed that low LF correlated with several CVD markers, including cIMT, augmentation index, AASI, and BP abnormalities on ABPM. In pediatric CKD, increased cIMT is used as a cardiovascular risk marker for large artery structure and is associated with high BP in children [37,38]. The augmentation index, which reflects arterial stiffness derived from the pressure waveform obtained from the ascending aorta, is elevated in children with CKD [39]. Similarly, the AASI, another marker of arterial stiffness, is associated with adverse cardiovascular events [29]. Prior research has also linked elevated AASI with high BP in pediatric CKD [28,40]. These findings suggest that low LF levels may be related to higher cardiovascular risk. 

Given LF’s known anti-inflammatory, antioxidant, and renoprotective effects, reduced plasma LF levels in CKD children with cardiovascular risk could indicate insufficient or impaired LF function. Alternatively, these low levels might signify a reduced capacity to prevent the progression of CVD later in life. To date, no data have indicated low LF levels in CKD patients with hypertension, as demonstrated in the current study. Animal models highlight the beneficial effects of LF in managing hypertension [41,42]. Given these properties of LF, our findings provide new insights into its potential protective role against hypertension associated with CKD.

Certain limitations warrant acknowledgment. First, the sample size, particularly for advanced stages of CKD, was relatively small, which may limit the ability to detect subtle differences between CKD stages and may not fully represent the broader population. Larger studies with more extensive cohorts are needed to validate our findings. Second, we studied the associations between LF and CVD markers between different stages of CKD but did not recruit normal controls. While the plasma LF levels in our study align with previous reports [34], it remains unclear whether LF levels differ between children with and without CKD. Further research is needed to clarify this relationship and to determine whether variations in LF levels could serve as a distinguishing marker for CKD in pediatric populations. Lastly, as this is a cross-sectional study, we cannot establish causality, but can only describe the observed associations. Additional longitudinal studies are needed to clarify the predictive capacity of LF in delineating the progression of CVD in children with CKD. Given that overt CVD is rare in children, tracking surrogate markers of CVD, such as cIMT, ABPM, and arterial stiffness index, over time is essential. Further research is necessary to elucidate LF’s role in CVD and to investigate the underlying mechanisms.

## 5. Conclusions

Data on the role of LF in pediatric CKD, particularly in its early stages, are limited. Cardiovascular risk markers can appear early in pediatric CKD, and our study found that low plasma LF concentrations were associated with several such markers, including cIMT, augmentation index, AASI, and abnormalities in ABPM. Although the mechanisms by which LF influences cardiovascular outcomes in pediatric CKD remain unclear, our findings suggest that LF could be a valuable biomarker for detecting subclinical CVD in children with CKD. Gaining a deeper understanding of LF’s impact on cardiovascular risk could enhance risk stratification and facilitate the early initiation of treatment, potentially improving outcomes for CKD children at risk of CVD.

## Figures and Tables

**Table 1:** Cohort characteristics of the study population (n = 102).

Characteristics	CKD Stage G1	CKD Stage G2–G4
	n = 73	n = 29
Age, years	9.7 (6.1–12.8)	10.9 (6.8–15.8)
Male	35 (46.6%)	22 (75.9%) *
CAKUT	45 (61.6%)	22 (75.9%)
Body height (percentile)	50 (25–85)	25 (15–75)
Body weight (percentile)	50 (25–85)	50 (15–85)
Body mass index (kg·m^−2^)	17.5 (15.4–20.5)	19 (15.4–21)
Systolic BP (percentile)	50 (50–95)	90 (50–99) *
Diastolic BP (percentile)	50 (50–95)	90 (50–95)
Blood urea nitrogen (mg/dL)	12 (10–14)	16 (13–24.5) *
Creatinine (mg/dL)	0.51 (0.43–0.6)	0.8 (0.65–1.16) *
eGFR (mL/min/1.73 m^2^)	107.3 (98.3–119.4)	72.7 (52–84.9) *
UTCR (mg/g)	65.7 (38.4–113.9)	152.2 (37.5–336.4)
Hemoglobin (g/dL)	13.5 (13–14.1)	13.5 (12.7–14.4)
Hematocrit (%)	40 (38.5–41.4)	40.3 (38.9–42.8)
Fasting glucose (mg/dL)	87 (84–92)	90 (82–92)
Total cholesterol (mg/dL)	172 (150–207)	185 (144–208)
Low-density lipoprotein (mg/dL)	94.5 (78–117.5)	100 (74–122)
Triglyceride (mg/dL)	67 (45–92)	66.5 (51–108.8)
Sodium (mmol/L)	140 (139–141)	140 (139–141)
Potassium (mmol/L)	4.3 (4.1–4.5)	4.5 (4.2–4.8)
Uric acid (mg/dL)	4.7 (4.2–5.5)	6.7 (5.6–7.5) *
Calcium (mg/dL)	9.9 (9.7–10.1)	10.1 (9.7–10.3)
Phosphate (mg/dL)	4.9 (4.6–5.2)	4.8 (4.5–5.2)
Hypertension (by office BP)	26 (35.6%)	13 (44.8%)
Overweight/obesity	24 (32.9%)	11 (37.9%)
Hyperlipidemia	23 (31.5%)	11 (37.9%)
Hyperuricemia	5 (6.8%)	15 (51.7%) *
Proteinuria	13 (17.8%)	12 (41.4)

Data presented as medians (IQR) or n (%). * *p* < 0.05 by the Mann–Whitney U-test.

**Table 2:** Comparison of cardiovascular assessments from 62 CKD children in the presence or absence of overweight/obesity.

CV Assessment	Normal	Overweight/Obesity
	n = 43	n = 19
Left ventricular mass (g)	73.8 (56.6–82.2)	101 (86.9–140) *
LVMI (g/m^2.7^)	25 (22–29.4)	32 (24.9–44.4) *
cIMT	0.35 (0.32–0.42)	0.38 (0.31–0.42)
Augmentation index	1.1 (−10.5–8.6)	−1.3 (−7–0.9)
PWV	3.9 (3.4–4.2)	4.3 (3.7–4.7) *
AASI	0.3 (0.15–0.44)	0.27 (0.2–0.49)

Data are medians (IQR). * *p* < 0.05 by the Mann–Whitney U-test.

**Table 3:** Correlation between plasma lactoferrin concentrations and cardiovascular assessments.

Cardiovascular Markers	Total (n = 62)	
	*r*	*p*
Left ventricular mass	0.185	0.15
LVMI	0.164	0.203
cIMT	−0.262	0.04 *
Augmentation index	−0.257	0.043 *
PWV	−0.008	0.954
AASI	−0.357	0.004 *

* *p* < 0.05 by Spearman’s correlation coefficient.

**Table 4:** Comparison of plasma LF concentrations in CKD children with normal and abnormal ABPM profiles.

ABPM Profile	*n*	Normal	n	Abnormal	
		Lactoferrin, ng/mL		Lactoferrin, ng/mL	*p* Value
24-h BP	54	1754 (1363–1874)	8	1312 (1025–1803)	0.11
Awake BP	53	1743 (1334–1873)	9	1306 (1013–1736)	0.062
Asleep BP	40	1798 (1586–1885)	22	1473 (1138–1767)	0.015
BP load	36	1777 (1392–1882)	26	1550 (1099–1861)	0.084
Night dipping	29	1845 (1730–1901)	33	1525 (1119–1791)	0.004
Total ABPM profile	23	1845 (1731–1914)	39	1566 (1138–1858)	0.006

Data are medians (range).

## Data Availability

The original data are saved at Kaohsiung Chang Memorial Hospital. Please contact the corresponding author for any inquiry.
